# Two new species of *Paratrigona* and the male of *Paratrigona ornaticeps* (Hymenoptera, Apidae)

**DOI:** 10.3897/zookeys.120.1732

**Published:** 2011-07-25

**Authors:** Victor H. Gonzalez, Terry L. Griswold

**Affiliations:** 1 USDA-ARS Bee Biology & Systematics Laboratory, Utah State University, Logan, Utah 84322-5310, USA

**Keywords:** Anthophila, Ecuador, Meliponini, Neotropics, Paraguay, stingless bees, taxonomy

## Abstract

Two distinctive new species of the Neotropical stingless bee genus *Paratrigona* Schwarz from Ecuador and Paraguay are described and figured. The Ecuadorian species, *Paratrigona scapisetosa* **sp. n.**,belongs to the *haeckeli-lineatifrons* group and is easily distinguished from its congeners by the unique shape and pubescence of the antennal scape, which is distinctly convex on its outer margin and bears thick, long, simple hairs along its inner margin. The Paraguayan species, *Paratrigona wasbaueri* **sp. n.**,belongs to the *lineata* group and is easily distinguished by the pattern of body pubescence in both sexes and male genitalic characters. The male of the Mesoamerican species *Paratrigona ornaticeps* (Schwarz) is described and figured. New geographical records for *Paratrigona impunctata* and *Paratrigona opaca*, and an updated key to the *haeckeli-lineatifrons* and *lineata* species groups are provided.

## Introduction

Stingless honey bees (Meliponini) are among the most commonly encountered bees in the tropics of the world. In the Americas, they are found from Mexico to Brazil and northern Argentina, inhabiting a diverse variety of ecosystems, including both humid and xeric lowlands to cloud forests and Páramos in the Andes reaching up to 4000 m of altitude (Gonzalez and Engel 2004; Nates-Parra 2006; Michener 2007; Camargo and Pedro 2008). Stingless bees are both ecologically and economically important. They are among the major pollinators of many native and cultivated tropical plants (e.g., Slaa et al. 2006); pollen, honey, and wax of some species have also been traditionally used by indigenous and non-indigenous peoples in rural areas across Latin America, thus representing an important source of income for these communities (e.g., Nates-Parra 2006; Michener 2007, and references therein).

There are about 500 species of stingless bees worldwide, most of them (80%) in the Western Hemisphere (Michener 2007; Camargo and Pedro 2008; Ascher and Pickering 2011). However, given the abundance of cryptic species, such numbers are probably underestimations of the true diversity of the group (Michener 2007). Despite several decades of taxonomic exploration, many stingless bees remain to be discovered and described. Work remains also in coming to a consensus on the generic and subgeneric classification of Meliponini. Some authors prefer to emphasize the differences, recognizing species or group of species with unusual characters at the generic or subgeneric rank, resulting in some 60 supraspecific taxa; others emphasize their relationships, placing such unusual taxa in more inclusive taxonomic units, with the result that only approximately 20 supraspecific taxa are recognized worldwide (reviewed in Michener 2007). For example, morphological and molecular data indicates that the two species placed by Moure (1951) in *Aparatrigona* Moure [*Paratrigona impunctata* (Ducke) and *Paratrigona isopterophila* (Schwarz)] are the sister group of *Paratrigona* Schwarz (Camargo and Moure 1994; Rasmussen and Cameron 2010). *Aparatrigona* is morphologically very similar to *Paratrigona* and has been treated as a subgenus of *Paratrigona* (Moure 1951), as a separated genus (Camargo and Moure 1994; Camargo and Pedro 2008), or synonymized with *Paratrigona* (Michener 2007). Similar cases occur with *Partamona* Schwarz and *Parapartamona* Schwarz, or with those taxa synonymized by Michener (2007) in *Trigonisca* Moure, which are treated at the generic level by Camargo and Pedro (2008). To some extent, the recognition of at least some of those unusual taxa at the generic or subgeneric level is a matter of opinion and good examples of lumper versus splitter views in bee systematics. Herein, we follow Michener’s classification for Neotropical Meliponini, except that we recognize at the generic level those taxa he placed as subgenera of *Trigona* Jurine and *Plebeia* Schwarz (Table 1). Recent molecular analyses (Rasmussen and Cameron 2007, 2010) indicate that these “subgenera” render *Trigona* and *Plebeia* paraphylectic as previously discussed by Michener (2007) based on morphology.

**Table 1.** Summary of generic and subgeneric classification of New World Meliponini including fossils. It follows Michener (2007) except by those taxa he placed as subgenera of *Trigona* and *Plebeia* which are herein recognized at the generic level. The distribution and approximate number of species are based on Michener (2007), Camargo and Pedro (2008), and Ascher and Pickering (2011). NA = North America; CA = Central America; SA = South America; † = Extinct, Miocene Dominican and Chiapas amber; ‡ = Extinct, Mesozoic New Jersey amber; * = one species is from Chiapas amber.

Taxa		Species	Distribution
Genus *Cephalotrigona* Schwarz		5	CA, SA
Genus *Cretotrigona* Engel‡		1	NA
Genus *Duckeola* Moure		2	SA
Genus *Frieseomelitta* Ihering		16	CA, SA
Genus *Geotrigona* Moure		21	CA, SA
Genus *Lestrimelitta* Friese		21	CA, SA
Genus *Melipona* Illiger		68	CA, SA
Genus *Meliwillea* Roubik, Segura & Camargo		1	CA
Genus *Nannotrigona* Cockerell		10	CA, SA
Genus *Nogueirapis* Moure		4*	CA, SA
Genus *Oxytrigona* Cockerell		11	CA, SA
Genus *Paratrigona* Schwarz		33	CA, SA
Genus *Paratrigonoides* Camargo & Roubik		1	SA
Genus *Partamona* Schwarz			
	Subgenus *Parapartamona* Schwarz	7	SA
	Subgenus *Partamona* Schwarz	34	CA, SA
Genus *Plebeia* Schwarz		40	CA, SA
Genus *Proplebeia* Michener†		4	CA
Genus *Scaptotrigona* Moure		21	CA, SA
Genus *Scaura* Schwarz		7	CA, SA
Genus *Schwarziana* Moure		2	SA
Genus *Tetragona* Lepeletier de Saint Fargeau & Serville		19	CA, SA
Genus *Tetragonisca* Moure		4	CA, SA
Genus *Trichotrigona* Camargo & Moure		1	SA
Genus *Trigona* Jurine		32	CA, SA
Genus *Trigonisca* Moure		39	CA, SA

The purpose of this paper is to describe two distinctive new species of *Paratrigona*, one each from Ecuador and Paraguay, and the male of the Mesoamerican species *Paratrigona ornaticeps* (Schwarz). We also provide new geographical records and an updated key to these species. Bees of this genus are small (4–6 mm in body length), often with a dull, dark brown to black integument and conspicuous, contrasting maculae on the head and mesosoma. There are 32 currently recognized species ranging from Mexico to southern Brazil, most of them known from the worker caste (Table 2). Although Meliponini taxonomy is based on workers, the study of the nesting behavior, males, and queens may provide additional characters useful in recognizing cryptic species and in phylogenetic analyses. Unfortunately, the nest and the male of *Paratrigona* are known for less than half of the species while the queen is only known for two species (Schwarz 1948; Camargo and Moure 1994; Nates-Parra et al. 1999; Fernández et al. 2010).

**Table 2.** Summary of currently included species in *Paratrigona* with information on the known sexes, nest and distribution. Sex/caste: ♀ = worker; ♂ = male; ‡ = queen. Nest: A = aerial; C = inside tree cavity; F = inside ant nest; S = subterranean; T = inside termite nest; - = unknown. The distribution and nesting site are based on Camargo and Moure (1994) and Camargo and Pedro (2008), with some modifications from Gonzalez and Vélez (2007), Fernández et al. (2010), and present study.

Species	Sex	Nest	Distribution
“guatemalensis species group”			
*Paratrigona anduzei* (Schwarz, 1943)	♀♂	A	Colombia, Venezuela
*Paratrigona eutaeniata* Camargo & Moure, 1994	♀♂‡	A	Colombia
*Paratrigona guatemalensis* (Schwarz, 1938)	♀	A	Costa Rica, Guatemala, Mexico
*Paratrigona lophocoryphe* Moure, 1963	♀♂	-	Colombia, Costa Rica, Panama
*Paratrigona rinconi* Camargo & Moure, 1994	♀	-	Colombia
“haeckeli-lineatifrons species group”			
*Paratrigona compsa* Camargo & Moure, 1994	♀	-	Brazil
*Paratrigona euxanthospila* Camargo & Moure, 1994	♀	-	Brazil
*Paratrigona femoralis* Camargo & Moure, 1994	♀	-	French Guiana
*Paratrigona guigliae* Moure, 1960	♀	-	Bolivia
*Paratrigona haeckeli* (Friese, 1900)	♀♂	-	Brazil, Peru
*Paratrigona lineatifrons* (Schwarz, 1938)	♀	T	Brazil
*Paratrigona melanaspis* Camargo & Moure, 1994	♀	-	Brazil
*Paratrigona onorei* Camargo & Moure, 1994	♀	-	Bolivia, Ecuador
*Paratrigona ornaticeps* (Schwarz, 1938)	♀♂	C	Costa Rica, Panama
*Paratrigona scapisetosa* Gonzalez & Griswold sp. n.	♀	-	Ecuador
*Paratrigona uwa* Gonzalez & Vélez, 2007	♀	-	Colombia
“impunctata species group”			
*Paratrigona impunctata* (Ducke, 1916)	♀♂	T	Brazil, Colombia, Ecuador, French Guiana, Guyana, Peru, Suriname
*Paratrigona isopterophila* (Schwarz, 1934)	♀♂	-	Colombia, Panama
“lineata species group”			
*Paratrigona crassicornis* Camargo & Moure, 1994	♀	-	Brazil
*Paratrigona incerta* Camargo & Moure, 1994	♀	-	Brazil
*Paratrigona glabella* Camargo & Moure, 1994	♀♂	-	Argentina, Bolivia
*Paratrigona lineata* (Lepeletier, 1836)	♀♂	S	Bolivia, Brazil
*Paratrigona nuda* (Schwarz, 1943)	♀	F	Bolivia, Peru
*Paratrigona prosopiformis* (Gribodo, 1893)	♀♂	-	Bolivia, Brazil, Colombia, Ecuador, Peru
*Paratrigona subnuda* Moure, 1947	♀♂	S	Brazil
*Paratrigona wasbaueri* Gonzalez & Griswold sp. n.	♀♂	-	Paraguay
“peltata-opaca species group”			
*Paratrigona catabolonota* Camargo & Moure, 1994	♀	-	Brazil
*Paratrigona lundelli* (Schwarz, 1938)	♀	-	Belize, Guyana
*Paratrigona myrmecophila* Moure, 1989	♀	F	Brazil
*Paratrigona opaca* (Cockerell, 1917)	♀♂	F	Colombia, Costa Rica, Ecuador, Guatemala, Mexico, Panama
*Paratrigona pacifica* (Schwarz, 1943)	♀♂‡	A	Bolivia, Brazil, Colombia, Ecuador, Peru
*Paratrigona pannosa* Moure, 1989	♀	-	Brazil, French Guiana
*Paratrigona peltata* (Spinola, 1853)	♀	-	Brazil
Incertae sedis			
*Paratrigona permixta* Camargo & Moure, 1994	♀	-	Venezuela

## Material and methods

Morphological terminology follows that of Michener (2007), while the format for the descriptions generally follows that used by Gonzalez and Vélez (2007), with variation in color in parentheses. Photomicrographs were taken using a Keyence® VHX-500F Digital Imaging System. Measurements were made with an ocular micrometer attached to a Leica MZ12 stereomicroscope. Measurements in descriptions are for the holotype, with values for paratypes in parentheses. The symbol for female and that word itself are used below for worker, not queen.

Institutional acronyms used herein are:

**BBSL**	U.S. National Pollinating Insects Collection, Bee Biology and Systematics Laboratory, Utah State University, Logan, UT, USA.

**DZUP**	Departamento de Zoologia, Universidade Federal do Paraná, Brazil.

**SEMC**	Snow Entomological Collection, Division of Entomology, University of Kansas Natural History Museum, Lawrence, Kansas, USA.

**USNM**	National Museum of Natural History, Washington, DC, USA.

## Systematics

### Tribe Meliponini Lepeletier de Saint Fargeau, 1836. Genus Paratrigona Schwarz, 1938

#### Paratrigona scapisetosa sp. n.

urn:lsid:zoobank.org:act:E7B29A75-9CEE-4245-877C-276952559457

http://species-id.net/wiki/Paratrigona_scapisetosa

Figs 1–4 

##### Holotype.

♀, Ecuador: Mor. [Morona] Santiago, Miazal, 50 km. SE Macas, 1-4/7-1-1993, 300 m., M & J. Wasbauer Coll. (BBSL).

##### Diagnosis.

**Figures 1–5.**
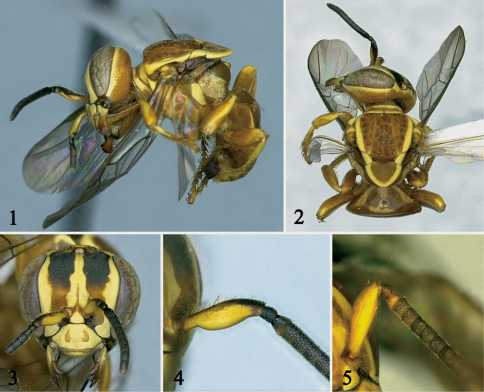
Female holotype of *Paratrigona scapisetosa* Gonzalez and Griswold, sp. n. (1–4), and *Paratrigona haeckeli* (5). **1–3** Lateral, dorsal, and frontal views, respectively **4, 5** Antennal scape in profile view.

This species belongs to the *haeckeli-lineatifrons* species group *sensu* Camargo and Moure (1994) recognized by the anterior margin of scutum rounded and the presence of maculations on frons, gena, mese- and metepisternum. It is most similar to *Paratrigona haeckeli* (Friese) sharing the maculations on face, thick antennal scape (1.4–1.5 times width of third flagellomere), and body predominantly ferruginous. It can be easily distinguished from that species and any other species of the genus by the distinctive shape and pubescence of the antennal scape. In *Paratrigona scapisetosa* the scape is distinctly convex on its outer margin, abruptly narrowed on basal one-fourth, and bears long (0.5 times width of third flagellomere), stiff, erect, simple hairs along its inner margin (Fig. 4). In *Paratrigona haeckeli* the scape is gently convex on its inner margin, straight or nearly so on its outer margin, and lacks long hairs (Fig. 5). Also, *Paratrigona scapisetosa* differs from *Paratrigona haeckeli* in the preoccipital margin laterally with few stiff, erect simple hairs as those on scape, and the propodeum yellow laterally (Fig. 1).

##### Description.

*Worker*: Total body length 4.3 mm; head width 1.7 mm; forewing length 3.5 mm. Head slightly wider than long; inner orbits of compound eyes converging below (Fig. 3); malar area subequal to width of third flagellomere; clypeus about twice as broad as long; interalveolar distance equal to median ocellar diameter, shorter (0.8x) than alveolar diameter; alveolocular distance 1.7 times alveolar diameter, 2.1 times interalveolar distance; interocellar distance about two times median ocellar diameter, 1.2 times longer than ocellocular distance; ocelloccipital distance about 1.3 times median ocellar diameter; scape 3.3 times longer than wide, 1.5 times broader than width of third flagellomere, distinctly convex on outer margin, abruptly narrowed on basal one-fourth; pedicel about as long as broad, slightly shorter than first flagellomere; flagellomeres slightly broader than long, except apical flagellomere much longer than broad (Fig. 4); compound eye 2.7 times longer than broad; gena 0.7 times narrower than compound eye in profile. Scutum with anterior margin rounded, 1.2 times broader than long, about twice as long as scutellum; scutellum 1.4 times broader than long, narrowly rounded on distal margin (long-paraboloid *sensu* Camargo and Moure 1994); hind tibia 2.6 times longer than broad; hind basitarsus about twice as long as broad, strongly projecting on posterodistal margin.

Integument in general finely and densely punctate, dull, as in other species of the genus; propodeum without impunctate midline.

Color predominantly ferruginous, except: black on upper two-thirds of frons, apex of scape, pedicel, flagellum; dark reddish brown on subantennal area, epistomal sulcus, malar area, mandible distally (darker on teeth), mesepisternum ventrally, hind tibia distally, outer surfaces of middle and hind basitarsi; yellow maculations as follows: mandible basally, labrum, scape, face (Fig. 3), gena and vertex with complete, thin band (at most as wide as median ocellar diameter), pronotum, pronotal lobe, anterior one-third of tegula, costal sclerite, mesepisternum laterally, metepisternum entirely, propodeum laterally, scutum laterally with broad band (as wide as median ocellar diameter), axilla, lateral and distal margins of scutellum with narrower band than on scutum, apices of middle and hind femora, outer surfaces of fore and middle tibiae with narrow band, hind tibia with band on posterior margin, fore and middle basitarsi basally. Wing membrane subhyaline with weak greenish and coppery reflections; veins and stigma dark brown (Figs 1, 2).

Body sparsely covered with minute, appressed, simple yellowish to whitish hairs except as follows: inferior margin of mandible, labrum, and hypostomal area with long (about as long as median ocellar diameter), erect hairs; scape with stiff, erect, simple hairs (0.5 times width of third flagellomere) along inner margin (Fig. 4); gena laterally with few stiff, erect hairs as those on scape; mesepisternum ventrally, middle and hind coxae, sterna with much longer, erect simple hairs (about twice as long as median ocellar diameter); sides of propodeum with dense, branched, semierect hairs (0.5 times median ocellar diameter) partially obscuring integument.

*Male*: Unknown.

*Queen*: Unknown.

##### Etymology.

The specific epithet is a combination of the Latin words “*scāpus*” and “*saetae*”, in reference to the distinctly long hairs along the inner margin of the antennal scape.

##### Comments.

An additional female specimen of *Paratrigona scapisetosa* from Sierra de Chiribiquete in Colombia (Department of Caquetá) has been examined by one of us (VHG) but it was not available for study. The propodeum laterally with yellow maculations of *Paratrigona scapisetosa* is a character only shared with *Paratrigona ornaticeps*.

#### Paratrigona wasbaueri sp. n.

urn:lsid:zoobank.org:act:EB384673-259D-46E3-B2F6-825BE6088FC2

http://species-id.net/wiki/Paratrigona_wasbaueri

Figs 6 19 

##### Holotype.

♀, Paraguay: San Pedro, Cororo-Rio Ypane, XII-5/9-1983, Malaise Trap, M. Wasbauer coll. (BBSL).

##### Paratypes.

54♀, 28♂, with same data as holotype but collected on November 24–30, and December 1–4 (BBSL, DZUP, SEMC).

##### Diagnosis.

**Figures 6–10.**
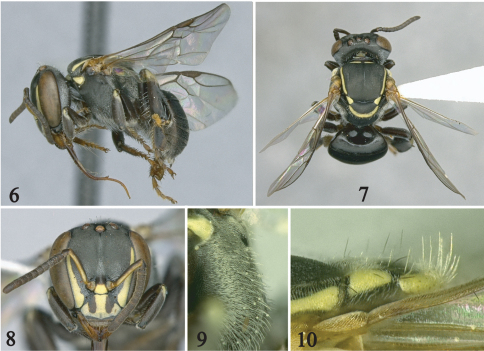
Female holotype of *Paratrigona wasbaueri* Gonzalez and Griswold, sp. n. **6–8** Lateral, dorsal, and frontal views, respectively **9** Mesepisternum dorsally in profile **10** Apex of scutum, axilla, and scutellum in profile.

This species belongs to the *lineata* species group *sensu* Camargo and Moure (1994) recognized by the anterior margin of scutum rounded and the absence of maculations on frons, gena, mese- and metepisternum (Figs 6–8). It is most similar to *Paratrigona lineata* (Friese) sharing the narrow antennal scape (narrower than width of third flagellomere), and the presence of hairs on the disc of scutellum distally and mesepisternum dorsally (Figs 9, 10). It can be easily distinguished from that species by the absence of hairs on the disc of the scutum in both sexes and the digitiform basal projection of the penis valve in the male (Figs 18, 19). The new species also resembles *Paratrigona glabella* Camargo and Moure and *Paratrigona incerta* Camargo and Moure in the absence of erect hairs on the scutum; however, in those species the mesepisternum lacks erect hairs dorsally and the erect hairs of the scutellum are either short (at most half median ocellar diameter) or restricted to its distal margin or inferior surface. Also, the basal projection of the penis valve of the male is short and pointed in *Paratrigona glabella* (male of *Paratrigona incerta* unknown), not digitiform as in *Paratrigona wasbaueri* (cf., Camargo and Moure 1994; fig 143).

**Figures 11–19.**
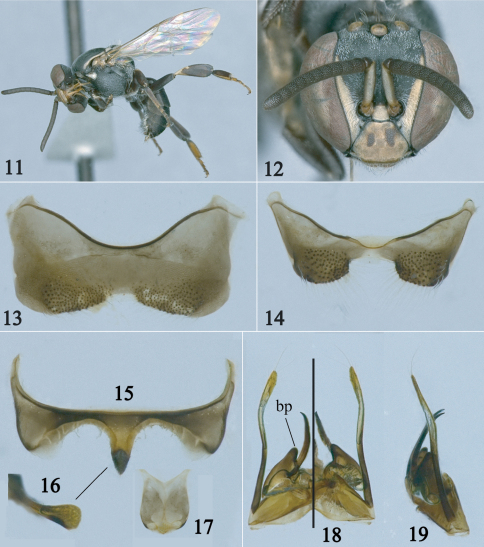
Male of *Paratrigona wasbaueri* Gonzalez and Griswold, sp. n. **11** Lateral habitus **12** Facial view **13** Fourth sternum **14** Fifth sternum **15** Sixth sternum **16** Apex of sixth sternum in profile **17** Seventh sternum **18** Genitalia in dorsal (left half) and ventral (right half) views **19** Genitalia in profile. **bp** = basal projection of penis valve.

##### Description.

*Worker*: Total body length 4.2 mm (3.8–5.2 mm); head width 1.6 mm (1.6–1.7 mm); forewing length 3.6 mm (3.6–3.8 mm). Head slightly wider than long; inner orbits of compound eyes converging below (Fig. 8); malar area about half width of third flagellomere; clypeus 1.8 times broader than long; interalveolar distance 1.2 times median ocellar diameter, about equal to alveolar diameter; alveolocular distance 1.3 times alveolar diameter; interocellar distance 2.1 times median ocellar diameter, 1.8 times longer than ocellocular distance; ocelloccipital distance about 1.3 times median ocellar diameter; scape 6.3 times longer than wide, narrower (0.8 times) than width of third flagellomere; pedicel about as long as broad, slightly shorter than first flagellomere; flagellomeres slightly broader than long, except apical flagellomere much longer than broad; compound eye 2.8 times longer than broad; gena 0.8 times narrower than compound eye in profile. Scutum with anterior margin rounded, slightly broader than long, 2.8 times longer than scutellum; scutellum about twice as long as broad, broadly rounded on distal margin (short-semicircular *sensu* Camargo and Moure 1994); hind tibia 2.6 times longer than broad; hind basitarsus about twice as long as broad, strongly projecting on posterodistal margin.

Integument in general finely and densely punctate, dull, as in other species of the genus; propodeum without impunctate midline.

Color black, except: light reddish brown on mandible (darker on teeth), tegula, margins of middle and posterior basitarsi, distitarsi; yellow maculations as follows: labrum, scape, face (Fig. 8), pronotum dorsally with medially interrupted band, pronotal lobe, scutum laterally with narrow band (about half width median ocellar diameter), axilla, lateral and distal margins of scutellum with broader band than on scutum, tibiae basally. Wing membrane hyaline with weak greenish and coppery reflections; veins and stigma dark brown (Figs 6, 7).

Body sparsely covered with minute, appressed, simple whitish hairs except as follows: inferior margin of mandible, labrum, vertex, hypostomal area, mesepisternum dorsally, distal margin of scutellum dorsally and ventrally, sides of propodeum with long (about as long as median ocellar diameter) erect hairs; mesepisternum ventrally, middle and hind coxae, sterna with much longer, erect simple hairs (about twice as long as median ocellar diameter); sides of propodeum with dense, branched, semierect hairs (0.5 times median ocellar diameter) partially obscuring integument; inner surface of tarsi with yellowish to ferruginous hairs.

*Male*: As in female except for longer body pubescence, brownish legs, and the following: Total body length 4.4–5.3 mm; head width 1.6 –1.7 mm; forewing length 3.7–3.8 mm. Inner orbits of compound eyes strongly converging below (Fig. 12); malar area linear; clypeus 1.3 times broader than long; interalveolar distance subequal to median ocellar and alveolar diameters individually; alveolocular distance about as long as alveolar diameter; interocellar distance about twice median ocellar diameter, 2.8 times longer than ocellocular distance; ocelloccipital distance about 0.7 times median ocellar diameter; scape 3.6 times longer than wide, slightly narrower than width of third flagellomere; pedicel longer than first flagellomere; first flagellomere about twice as broad as long, 0.5 times shorter than second flagellomere; gena about half width of compound eye in profile. Hind tibia about 3 times longer than broad; hind basitarsus 3 times longer than broad. Genitalia and associated sterna as in Figs 13–19.

*Queen*: Unknown.

##### Etymology.

The specific epithet is a patronym honoring Marius Wasbauer, friend and colleague, who has contributed much of the material we have studied, including the species described in this paper.

##### Comments.

In most paratypes the yellow maculations are pale, discolored, most likely due to preservation in alcohol for an extended period.

#### Paratrigona ornaticeps

(Schwarz, 1938)

http://species-id.net/wiki/Paratrigona_ornaticeps

Figs 20–27

Trigona (Paratrigona) opaca ornaticeps  Schwarz, 1938

##### Diagnosis.

The female of this species can be separated from other species of the *haeckeli-lineatifrons* group by the following combination of characters: frons with broad and long inverted Y-shaped maculation; paraocular area with complete, long, broad maculation; supraclypeus yellow; and clypeus with broad, inverted T-shaped maculation (cf., Camargo and Moure 1994; fig 115). *Paratrigona ornaticeps* can be confused with *Paratrigona uwa* Gonzalez and Vélez and *Paratrigona compsa* Camargo and Moure by the shape of the facial maculations but in those species they are much narrower and the metasoma is ferruginous (cf., Camargo and Moure 1994; fig 118; Gonzalez and Vélez 2007; fig 1).

The male of *Paratrigona ornaticeps* can be separated from that of *Paratrigona haeckeli*, the only other male known of the *haeckeli-lineatifrons* group (Table 2), by the sixth sternum, which has a broader midapical projection, and the shape of the gonostylus, which is more strongly curved apically in profile (compare Figs 26 and 27 with Camargo and Moure 1994; figs 130, 132).

**Figures 20–27.**
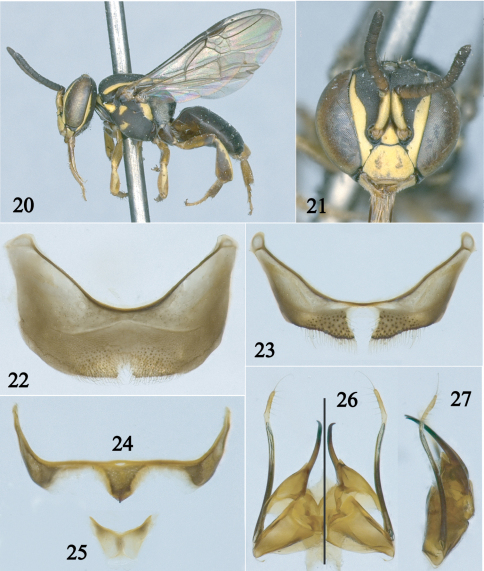
Male of *Paratrigona ornaticeps* (Schwarz). **20** Lateral habitus **21** Facial view **22** Fourth sternum **23** Fifth sternum **24** Sixth sternum **25** Seventh sternum **26** Genitalia in dorsal (left half) and ventral (right half) views **27** Genitalia in profile.

##### Description.

*Male*: Total body length 4.7–5.1 mm; head width 1.6 –1.7 mm; forewing length 3.7–3.8 mm. Head slightly wider than long; inner orbits of compound eyes strongly converging below (Fig. 21); malar area very short, about one-fourth width of third flagellomere; clypeus 1.5 times broader than long; interalveolar distance slightly shorter (0.9x) than median ocellar and alveolar diameters individually; alveolocular distance 1.3 times alveolar diameter, 1.2 times interalveolar distance; interocellar distance 1.5 times median ocellar diameter, 2.7 times longer than ocellocular distance; ocelloccipital distance about 0.7 times median ocellar diameter; scape 2.8 times longer than wide, slightly broader than width of third flagellomere; pedicel about as long as broad, slightly longer than first flagellomere; first flagellomere 1.5 times broader than long, 0.7 shorter than second flagellomere, remaining flagellomeres slightly broader than long, except apical flagellomere much longer than broad; compound eye 2.6 times longer than broad; gena about half width of compound eye in profile. Scutum with anterior margin rounded, 1.1 times broader than long, 2.3 times longer than scutellum; scutellum 1.5 times broader than long, broadly rounded on distal margin (long-paraboloid *sensu* Camargo and Moure 1994); hind tibia about 3 times longer than broad; hind basitarsus 2.6 times longer than broad. Genitalia and associated sterna as in Figs 22–27.

Integument in general finely and densely punctate, dull, as in other species of the genus; propodeum with faint midline.

Color predominantly black, except: brownish on anterior surface of flagellum, tegula, legs (legs light yellow to ferruginous with pale maculations); yellow maculations as follows: mandible, labrum, anterior surface of scape, face as in Fig. 21 (frons sometimes maculate along middle line, never with inverted broad Y-shaped maculation as in female), gena basally with thin band (less than half median ocellar diameter), vertex with small spot laterally, pronotum, pronotal lobe, tegula with small spot on anterior half, costal sclerite, mesepisternum laterally with spots, metepisternum almost entirely, scutum laterally with broad band (about half median ocellar diameter), axilla, lateral and distal margins of scutellum with narrower band than on scutum, apices of middle and hind femora, outer surfaces of fore and middle tibiae with narrow band, hind tibia with band on posterior margin, fore and middle basitarsi basally. Wing membrane subhyaline with weak greenish and coppery reflections; veins and stigma dark brown (Fig. 20).

Body sparsely covered with minute, appressed, simple yellowish to whitish hairs except as follows: vertex, hypostomal area, middle trochanter with long (about half median ocellar diameter), erect hairs; hind coxa, seventh tergum with much longer erect hairs (at least as long as median ocellar diameter); sides of propodeum with dense, branched, semierect hairs (about 0.5 times median ocellar diameter) partially obscuring integument.

##### Material examined.

(*n* = 33♀, 39♂) Costa Rica: 1♀, 1♂, Turrialba, VII-15-19-65, P.J. Spangler; 2♀, 5♂, S.J. [San José], San Isidro General, Feb. 1993, F. D. Parker; 1♀, 13♂, Alaj. [Alajuela], 20 km S Upala, collected on 1–10 April, 20–31 March, 22–31 May, 3–21 June 1991, and 1–10 August 1991, F. D. Parker; 25♀, 18♂, Alaj., Bijagua, 20 km S Upala, collected on 6–29 January, 12 February, 5–28 March, 10 April–6 May, 1–11 June, 1–15 July, 1–12 Aug, 1–15 Sep, 9–19 October, 1–20 November, and 25–30 December 1991, F. D. Parker; 4♀, Cart. [Cartago], Turribalba, CATIE, 26–29 June 1986, W. Hanson, G. Bohart ; 2♂, Guan [Guanacaste], 14 km S Cañas, collected on 17 March, 11–12 June 1991, F. D. Parker (BBSL, USMN).

##### Comments.

Unlike the female, the yellow maculations in the male are usually reduced on gena, vertex, pronotum, frons, and sides of propodeum.

#### Paratrigona impunctata

(Ducke)

http://species-id.net/wiki/Paratrigona_impunctata

Melipona impunctata Ducke 1916: 101 [♀].

##### New records.

(*n* = 5♀, 2♂) Ecuador: 2♀, 2♂, Napo Province, Huahua Sumaco, km. 45 on Hollin-Loreto road, XII-16-20, 1989, Malaise trap, M. & J. Wasbauer. Collrs; 1♀, Misahualli nr. Tena, 3–8 Oct., 1999, Steven R. Keller; 1♀, Yasuni Res. Sta. 19–30 Oct. 1998, W.J. Hanson, 250 m / 6°36'W, 0°38'S; *idem*, 1♀, 30 Sept.-11 Oct. 2002, C. Brammer, 250 m M.T [Malaise trap]., 0°40.566'S, 076°23.851W (BBSL).

##### Comments.

These new records expand the distribution of this species from its previously known range: Brazil, Colombia, French Guiana, Guyana, Peru, and Suriname (Table 2).

#### Paratrigona opaca

(Cockerell)

http://species-id.net/wiki/Paratrigona_opaca

Trigona opaca Cockerell 1917: 126 [♂].

##### New record.

Ecuador: 3♀, 5♂, Napo Province, Huahua Sumaco, km. 45 on Hollin-Loreto road, XII-20, 1989, Malaise trap, M. & J. Wasbauer, H. Real (BBSL).

##### Comments.

This new record expands the range of this species from its previously known distribution: Colombia, Costa Rica, Guatemala, Mexico, and Panama (Table 2).

### Key to species of the haeckeli-lineatifrons and lineata species groups of Paratrigona (workers)

**Table:** 

1	Maculations absent on frons, gena (except for some small spots in some specimens of *Paratrigona subnuda*), mesepisternum, and metepisternum (*lineata* species group)	2
–	Maculations present on frons, gena, mesepisternum, and metepisternum (*haeckeli-lineatifrons* species group)	9
2(1)	Antennal scape broad, about 1.3 times width of third flagellomere; submarginal angle (between Rs and Rs+M) obtuse (>100°); malar area longer than half width of third flagellomere	*Paratrigona prosopiformis* (Gribodo)
–	Antennal scape, at most, slightly broader than width of third flagellomere; submarginal angle acute (<90°); malar area narrower than half width of third flagellomere	3
3(2)	Scutellum short-paraboloid, length about 0.55 times its width; paraocular band usually sinuous at the level of antennal alveolus	*Paratrigona nuda* (Schwarz)
–	Scutellum short-semicircular, length between 0.50 and 0.51 times its width; paraocular band not sinuous at the level of antennal alveolus	4
4(3)	Scape narrower than width of third flagellomere	5
–	Scape about as broad as, or broader than, width of third flagellomere	8
5(4)	Disc of scutum with distinctly long, erect, simple hairs	*Paratrigona lineata* (Lepeletier)
–	Disc of scutum without long, erect, simple hairs	6
6(5)	Mesepisternum dorsally with long, erect, simple hairs (Fig. 9); scutellum dorsally with long (about as long as median ocellar diameter), erect, simple hairs on distal half (Fig. 10) (Paraguay)	*Paratrigona wasbaueri* sp. n.
–	Mesepisternum dorsally without long, erect, simple hairs; hairs on scutellum short (at most half median ocellar diameter) or absent	7
7(4)	Scutellum with erect hairs on distal margin and some on dorsal surface; face with pale yellow maculations; northern Argentina to Bolivia	*Paratrigona glabella* Camargo & Moure
–	Scutellum with erect hairs on inferior surface only, absent on distal margin; face with bright yellow maculations (northern Minas Gerais, Brazil)	*Paratrigona incerta* Camargo & Moure
8(4)	Scape distinctly broader (1.2–1.3 times) than width of third flagellomere (Pará, Amazonas, Brazil)	*Paratrigona crassicornis* Camargo & Moure
–	Scape about as broad as or slightly broader than width of third flagellomere (southeastern Brazil)	*Paratrigona subnuda* Moure
9(1)	Scape thick, about 1.5 times width of third flagellomere; body ferruginous	10
–	Scape, at most, slightly broader than width of third flagellomere; body black, especially head and mesosoma	11
10(9)	Scape distinctly convex on its outer margin, abruptly narrowed on basal one-fourth, inner margin straight or nearly so, with long (0.5 times width of third flagellomere), stiff, erect, simple hairs (Fig. 4)	*Paratrigona scapisetosa* sp. n.
–	Scape straight or nearly so on its outer margin, not abruptly narrowed on basal one-fourth, inner margin gently convex, without long hairs (Fig. 5)	*Paratrigona haeckeli* (Friese)
11(9)	Hind femur with superior margin protuberant on basal third	12
–	Hind femur unmodified, without superior margin protuberant on basal third	13
12(11)	Paraocular band broad, 1.4 times broader than width of third flagellomere, inferiorly filling the space between the compound eye and epistomal sulcus to the level of the tentorial pit	*Paratrigona euxanthospila* Camargo & Moure
–	Paraocular band narrow, about as broad as width of third flagellomere, inferiorly not filling the space between the compound eye and epistomal sulcus to the level of the tentorial pit	*Paratrigona femoralis* Camargo & Moure
13(11)	Maculations absent or reduced on inferior paraocular area and clypeus; paraocular band present above superior tangent of antennal alveolus; frontal maculation separated in two small spots, one below median ocellus, the other in the middle of frons; mesepisternum dorsally with spot as large as that on pronotal lobe; malar area short, slightly longer than half width of third flagellomere	*Paratrigona melanaspis* Camargo & Moure
–	Maculations of face not reduced on clypeus and supraclypeus; paraocular band reaching inferior tangent of antennal alveolus; frontal maculation usually complete; mesepisternum dorsally with or without spot; malar area variable in length	14
14(13)	Antennal scape robust, slightly broader than width of third flagellomere, black or with small basal spot; clypeus with reduced maculations, at most yellow along middle line; paraocular band narrower than width of third flagellomere, not reaching epistomal sulcus; mesepisternum black or with small spot; malar area short, slightly shorter than width of third flagellomere	*Paratrigona onorei* Camargo & Moure
–	Antennal scape slender, at most as broad as width of third flagellomere, maculate on anterior face; clypeus with well developed maculations, not restricted to middle line; paraocular band of variable width, reaching epistomal sulcus; mesepisternum with large spot; malar area variable in length	15
15(14)	Paraocular band narrow, slightly broader than half width of third flagellomere, about the same width along entire length; genal band narrow, not reaching vertex	*Paratrigona lineatifrons* (Schwarz)
–	Paraocular band broad, 1.4 times broader than width of third flagellomere wider in the middle or below antennal alveolus; genal band complete, reaching vertex and joining paraocular band or slightly interrupted	16
16(15)	Paraocular band very broad inferiorly, occupying space between compound eye and epistomal sulcus below tentorial pit	*Paratrigona ornaticeps* (Schwarz)
–	Paraocular band narrowed inferiorly, not occupying space between compound eye and epistomal sulcus below tentorial pit	17
17(16)	Malar area about as long as, or slightly longer than, width of third flagellomere; frontal maculation widest in the middle, just above bifurcation; clypeus yellow along distal margin (Andes, Colombia)	*Paratrigona uwa* Gonzalez & Vélez
–	Malar area length about 2/3 width of third flagellomere; frontal maculation not distinctly broad above bifurcation; clypeus entirely yellow, at most, darkened on disc (Amazonas, Brazil)	*Paratrigona compsa* Camargo & Moure

## Supplementary Material

XML Treatment for Paratrigona scapisetosa

XML Treatment for Paratrigona wasbaueri

XML Treatment for Paratrigona ornaticeps

XML Treatment for Paratrigona impunctata

XML Treatment for Paratrigona opaca
